# A vision transformer-radiomics approach for enhanced chemotherapy outcome prediction in ovarian cancer

**DOI:** 10.3389/fradi.2026.1702977

**Published:** 2026-03-17

**Authors:** Neman Abdoli, Patrik Gilley, Ke Zhang, Youkabed Sadri, Theresa Thai, Yong Chen, Lauren Dockery, Kathleen Moore, Robert Mannel, Yuchen Qiu

**Affiliations:** 1 School of Electrical and Computer Engineering, University of Oklahoma, Norman, OK, United States; 2 Stephenson School of Biomedical Engineering, University of Oklahoma, Norman, OK, United States; 3 Department of Radiology, University of Oklahoma Health Sciences Center, Oklahoma City, OK, United States; 4 Department of Radiation Oncology, University of Oklahoma Health Sciences Center, Oklahoma City, OK, United States; 5 Department of Obstetrics and Gynecology, University of Oklahoma Health Sciences Center, Oklahoma City, OK, United States

**Keywords:** chemotherapy response prediction, foundation models, ovarian cancer, radiomics, transfer learning, vision transformers

## Abstract

**Introduction:**

Early prediction of chemotherapy response in ovarian cancer patients is essential for enabling personalized treatment strategies and improving clinical outcomes. However, this prediction remains challenging due to the high heterogeneity of tumor biology, patient-specific factors, and treatment regimens. Recent advances in imaging biomarkers derived from both radiomics and advanced deep learning methods offer promising tools for characterizing tumor phenotypes and predicting treatment outcomes.

**Methods:**

In this retrospective study, pre-treatment CT scans from 182 ovarian cancer patients were analyzed. Three categories of imaging features were extracted: handcrafted radiomics descriptors, embeddings from a pretrained Vision Transformer (ViT), and embeddings from MedSAM, a medical foundation model adapted for segmentation. All features were standardized and subjected to least absolute shrinkage and selection operator (LASSO) regression for feature selection. Support vector machine (SVM) classifiers were trained to predict 6-month progression-free survival (PFS). Model performance was evaluated using cross-validated metrics including area under the receiver operating characteristic curve (AUC) and classification accuracy.

**Results:**

The combined ViT and MedSAM embedding model achieved the highest AUC of 0.924 ± 0.032. Integration of all three feature groups (radiomics, ViT, and MedSAM) yielded a comparable AUC of 0.924 ± 0.037 and the highest classification accuracy of 0.831 ± 0.042.

**Conclusion:**

These findings demonstrate that integrating complementary imaging representations enhances chemotherapy response prediction. The combination of transformer-based embeddings and radiomics features provides rich, task-specific tumor characterization from CT imaging and supports the development of precision oncology decision tools.

## 1 Introduction

Ovarian cancer (OC) is one of the most lethal gynecological malignancies (1). Most ovarian carcinoma cases are diagnosed at advanced stages due to the difficulty in early detection, which increases the risk of metastatic tumor development on multiple organs (2). Following primary cytoreduction, the standard treatment involves chemotherapy, directed at eliminating residual cancer cells and decreasing the risk of recurrence. Clinically, response to treatment is different among the patients due to the complexity of disease, particularly cellular and molecular heterogeneities of OC tumor (3). Therefore, predicting treatment effectiveness in the initial stages of therapy can improve the precision of patient selection for tailored treatment approaches, notably chemotherapy (4). A limitation in early prediction of treatment response for OC is the absence of biomarkers capable of reliably predicting chemotherapy efficacy (5, 6). Certain biomarkers, like cancer antigen 125 (CA125) and human epididymis protein 4 (HE4), are associated with patient response to chemotherapy (7, 8). However, their accuracy in predicting chemotherapy response varies among patients with different epidemiological and clinical features (9). Medical imaging modalities offer a non-invasive tool that can capture comprehensive data characterizing tumor heterogeneity. Among different imaging modalities, computed tomography (CT) is a widely utilized method for assessing therapy response due to its general availability and user-friendly nature (10). Tumor heterogeneity on CT images can be quantified using texture analysis which extracts spatial information that may not be perceptible to the naked eye (10).

Radiomics refers to the extraction and analysis of large amounts of quantitative imaging features with high throughput from two-dimensional and three-dimensional medical images (11). These features comprise mathematical descriptions of various visual properties observed in the images, encompassing attributes such as shape, texture, and density. Radiomics provides an objective method to assess the tumor microenvironment, capturing information that is not perceptible to the human eye (12). Several studies have been conducted to identify quantitative imaging markers related to treatment response using radiomics features (13–16). However, a major limitation of radiomics is its reliance on predefined, low-order features based on heuristic knowledge (17). Extracting higher-level, more advanced features and integrating them into the radiomics framework could enhance predictive power.

Deep learning (DL), including recent advancements such as vision transformers (ViT), and medical foundation models, has emerged as a prominent method for various image processing tasks, gaining increasing attention (18). These models have been utilized to extract information of tumor phenotype and micro-environment in detection, prognosis, and treatment outcome prediction studies from medical images (19). In the medical field, where datasets are often limited, a pretrained DL model can be applied to the downstream tasks. This practice, known as “transfer learning”, has been proven to be effective in many cases, such as predicting survival among lung adenocarcinoma patients and rectum toxicity in cervical cancer radiotherapy (20, 21). Similarly, deep learning models trained on medical images, such as histopathology images, can be employed to predict treatment response in OC (22, 23). However, there are limited existing studies rely on multi-method features that explore the full potential of integrating radiomics with high-level deep embeddings (24). Furthermore, the relative contributions of models pretrained on natural images vs. those fine-tuned on medical data remain insufficiently studied. There is a need for further investigation into how these different pretrained models complement radiomics features, and whether specific combinations of representations can enhance predictive performance in a synergistic manner.

Integrating advanced deep learning techniques with radiomics has the potential to overcome the limitations of traditional handcrafted features by leveraging rich, high-level embeddings. In this study, we explore the combination of radiomic features with deep representations extracted from ViT and MedSAM encoders, a medical image segmentation foundation model. We develop and evaluate multi-representation predictive models that integrate these diverse feature sets to predict chemotherapy response in OC treatment. This integrated approach aims to provide a more comprehensive representation of tumor characteristics and treatment dynamics, ultimately paving the way for more accurate and personalized cancer therapies. To the best of our knowledge, this is the first study to systematically explore the integration of radiomics and foundation model features for chemotherapy response prediction.

## 2 Materials and methods

### 2.1 Database

**Figure 1:**
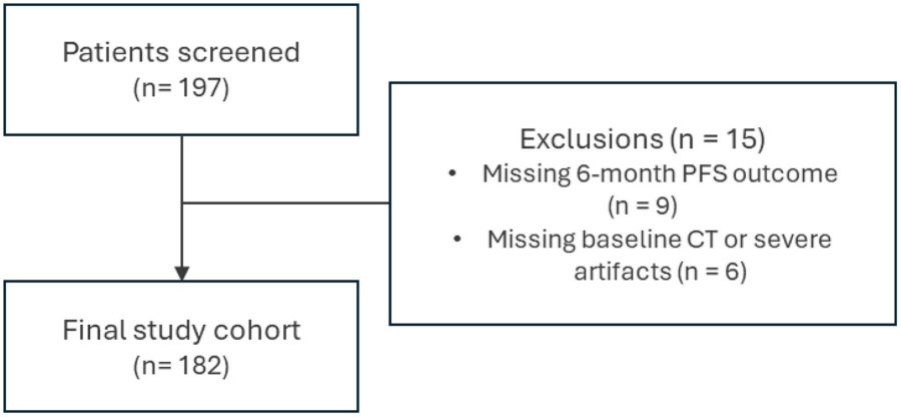
Cohort selection flow diagram.

In our study, we used an imaging dataset from 197 patients with OC treated at the University of Oklahoma health science center (OUHSC). These images were retrospectively collected from patients with advanced-stage OC, presenting with recurrent peritoneal or tubal carcinoma. Patients with pre-treatment CT scans and known 6-month PFS were included and cases without imaging or follow-up data were excluded (Figure 1). The study population included participants with various histological subtypes, such as endometrioid, serous, clear cell, and mucinous carcinomas. For each patient, radiologists examined the pre-therapy CT images and annotated all metastatic tumors, resulting in one to five marked lesions depending on the individual's disease burden. Following diagnosis, patients underwent systemic chemotherapy treatment post primary cytoreduction. Imaging procedures followed routine clinical protocols using GE LightSpeed VCT (64-slice) or GE Discovery 600 (16-slice) machines, with tube currents ranging from 100 to 600 mA based on patient size. Clinical outcome data included 6-month progression-free survival (PFS), an endpoint frequently adopted in ovarian cancer phase II/III trials to enable early assessment of therapeutic benefit and support timely treatment adaptation. Several studies have demonstrated that improvements in 6-month PFS are associated with longer-term survival and are used to inform go/no-go decisions and regulatory evaluation in clinical trial settings (25, 26). Of the 182 patients included in the final cohort, 124 showed positive 6-month PFS (responder), while 58 showed no or minimal response (non-responder). Our study was approved by the institutional review board at the university (IRB13649). Table 1 presents the detailed demographic characteristics of the patients cohort in this study.

**Table 1:** Summary of demographic and clinical characteristics.

Characteristic	PFS (6-month)		*p*-value
	Responder	Non-responder	
Number of patients	124	58	
Age	63 ± 10	61 ± 10	0.24
Number of tumors	264	131	
Average tumor diameter (mm)	31.3 ± 19.3	31.5 ± 16.4	0.94

### 2.2 Image feature computation

Before feature extraction, CT volumes were first resampled to isotropic voxel spacing to ensure consistent spatial resolution across patients. A soft-tissue HU window (WL = 40, WW = 400) was then applied to enhance lesion visibility, and the processed slices were subsequently linearly scaled to 8-bit grayscale and exported as PNG images without further normalization for standardized handling. These windowed images were used for all subsequent segmentation and feature extraction steps. Following this, we adopted a tumor segmentation method to segment regions of interest (ROIs) from CT images (27). This method included two key steps. First, an experienced radiologist annotated the largest tumor area on the CT images using the RECIST criteria (28). Then, a semi-automated hybrid algorithm was applied to segment the tumor. This algorithm combines a topographic region growth approach with adaptive thresholds and dynamic edge tracking, ensuring accurate identification of the metastatic tumor slice with the largest area. We used only the 2D slice with the largest tumor cross-section for all subsequent feature extraction to ensure consistency and minimize variability. Despite the algorithm's high performance, the inherent heterogeneity of metastatic tumors may occasionally result in suboptimal segmentation outcomes. To address this, we made manual corrections to refine the segmentation results as needed. The entire segmentation process was implemented as an ImageJ plugin (29) with a user-friendly graphical user interface (GUI), facilitating visualization and manipulation of 2D tumor contours.

Based on the extracted image ROI, we computed handcrafted features and deep learning-based embeddings. In order to extract hand crafted features, we employed established radiomics methodologies to derive a comprehensive set of quantitative imaging features characterizing tumor texture, shape, and intensity (11). In addition, we employed state-of-the-art pretrained ViTs and foundation models to extract high-level embeddings that capture intricate spatial information within the images (18, 30).

#### 2.2.1 Radiomics features

Radiomics feature computation was conducted using the pyradiomics platform (31), adhering to the definitions established by the imaging biomarker standardization initiative (IBSI) (32). Initially, the 2D tumor images underwent a filtering process, including exponential, gradient magnitude, local binary pattern (LBP), logarithm, square, square root, and wavelet (Coif1) filters. Subsequently, radiomics features were computed on both the processed images and the original image, encompassing geometric, density, and texture features. Geometric features were exclusively extracted from the original image, offering insights into tumor morphology, while density features described the intensity distribution of pixels within the segmented region. Texture features are derived by matrices such as gray level co-occurrence matrix (GLCM) (33), gray level dependence matrix (GLDM) (34), gray level run length matrix (GLRLM) (35), gray level size zone matrix (GLSZM) (36), and neighboring gray tone difference matrix (NGTDM) (34). These features capture spatial patterns and variations in pixel intensities. In total, 1,218 features were computed for the 2D radiomics feature pool. From each image type, 18 first-order features, 24 GLCM, 14 GLDM, 16 GLRLM, 16 GLSZM, and 5 NGTDM features were extracted, along with 9 geometric features extracted from the original image.

#### 2.2.2 Deep learning features

##### 2.2.2.1 Embeddings from vision transformers

**Figure 2:**
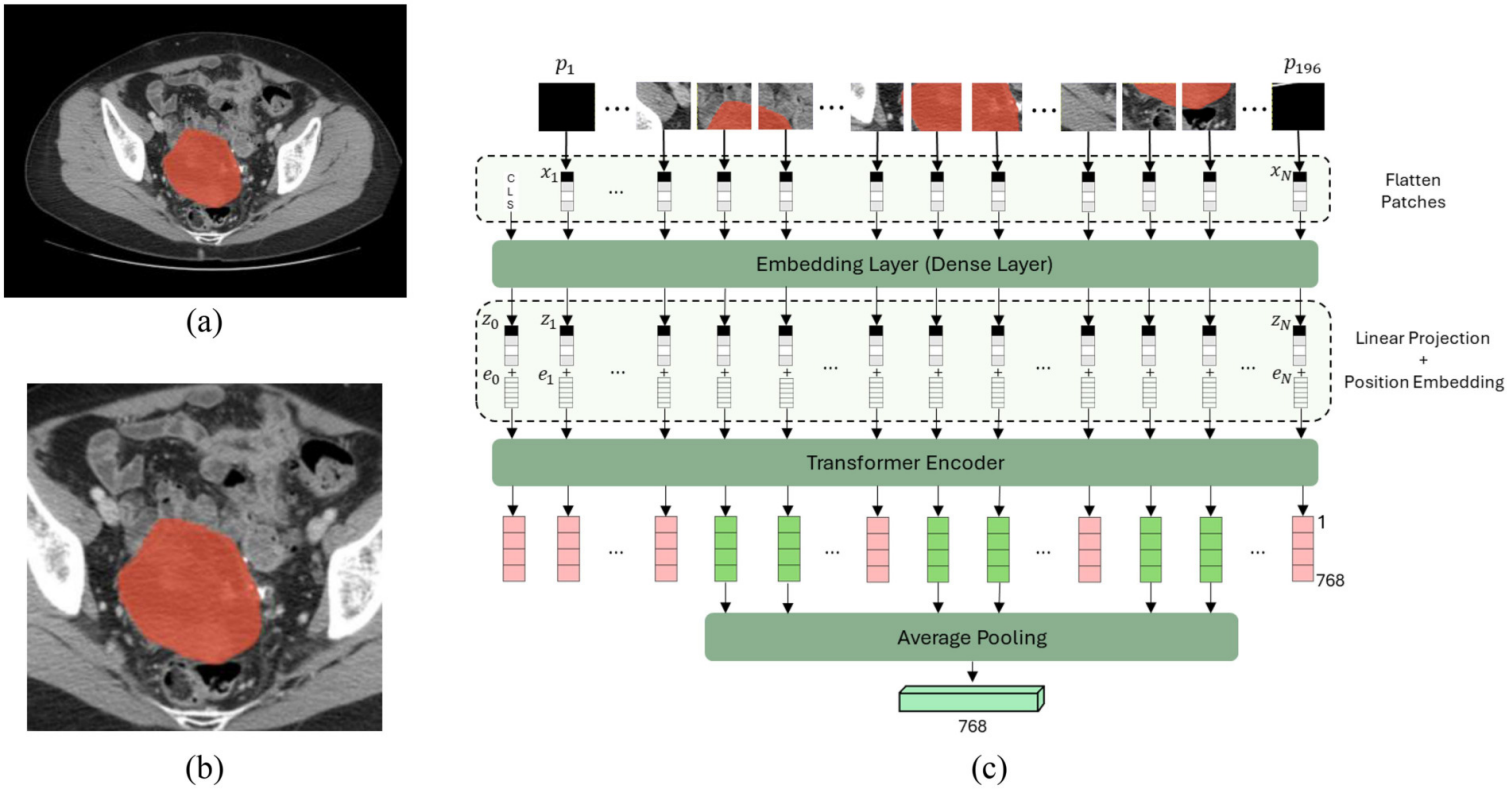
Vision transformer-based feature extraction process. **(a)** CT image with the identified and highlighted tumor. **(b)** Extracted region of interest (ROI) around the tumor. **(c)** Block diagram illustrating the ViT-based feature extraction.

In this study, we utilized the embeddings from the ViT base model (18). The ViT architecture was originally pretrained on images from ImageNet dataset with a resolution of 224*224 for image classification purposes (37). These embeddings encode high-level representations that reflect detailed characteristics of image intensity, texture, and edge information (38). For this reason, each preprocessed grayscale CT image was replicated across three channels and cropped to a 224 × 224 region centered on the ROI. The resulting images were then divided into 14 × 14 non-overlapping patches (Figure 2). Afterwards, the patches were flattened and linearly embedded into 1D vectors. Learnable positional encodings were added to these vectors, and a learnable [CLS] token was prepended to the sequence. In the figure, let $p_{1}$, …, $p_{196}$ denote the patches of an input image. The transformer encoder comprises a stack of multiple blocks, each composed of multi-head attention and feed-forward neural networks. At its core, the transformer encoder leverages the self-attention mechanism, enabling the model to assess the importance of different patches in the sequence during data processing (18). This self-attention mechanism effectively captures global contextual information, facilitating the learning of long-range dependencies and relationships among image patches. Finally, to refine the feature representation, we averaged the feature vectors corresponding to patches with tumor and excluded those from unaffected areas, resulting in a 1D feature vector with a size of 768 dimensions.

##### 2.2.2.2 Deep embeddings from MedSAM

MedSAM (30) is a foundation model built upon the Segment Anything Model (SAM) (39). It is fine-tuned on more than 1.5 million medical images, including more than 500,000 CT scans, each paired with segmentation masks to capture domain-specific visual representations. The model architecture consists of a transformer-based image encoder, a prompt encoder, and a mask decoder. The image encoder is based on the ViT-Base model, which was pretrained using masked autoencoding (40). Given the strong representational capacity of its ViT encoder, which is trained on large-scale medical datasets with extensive CT coverage, we used MedSAM as a feature extractor in this study. This allowed us to capture high-level semantic features relevant to our prediction task.

In our study, we resized each 2D CT image from 512 × 512 to 1,024 × 1,024 pixels to match the MedSAM input format. Embeddings were then extracted from non-overlapping 16 × 16 patches of the image. To ensure focus on tumor-relevant regions, we adopted a patch selection strategy similar to that used in ViT, retaining only the embedding vectors related to patches that contained at least 50% tumor area. Finally, average pooling was applied to generate a fixed-length feature vector (of size 768) for each tumor.

When multiple lesions were present in a patient, radiomics and deep features were computed separately for each lesion. To generate a patient-level feature representation, lesion-level feature vectors were aggregated using average pooling, producing a fixed-size feature vector per patient regardless of lesion count. The resulting patient-level vectors were used as input for model training and outcome prediction.

### 2.3 Machine learning model development

**Figure 3:**
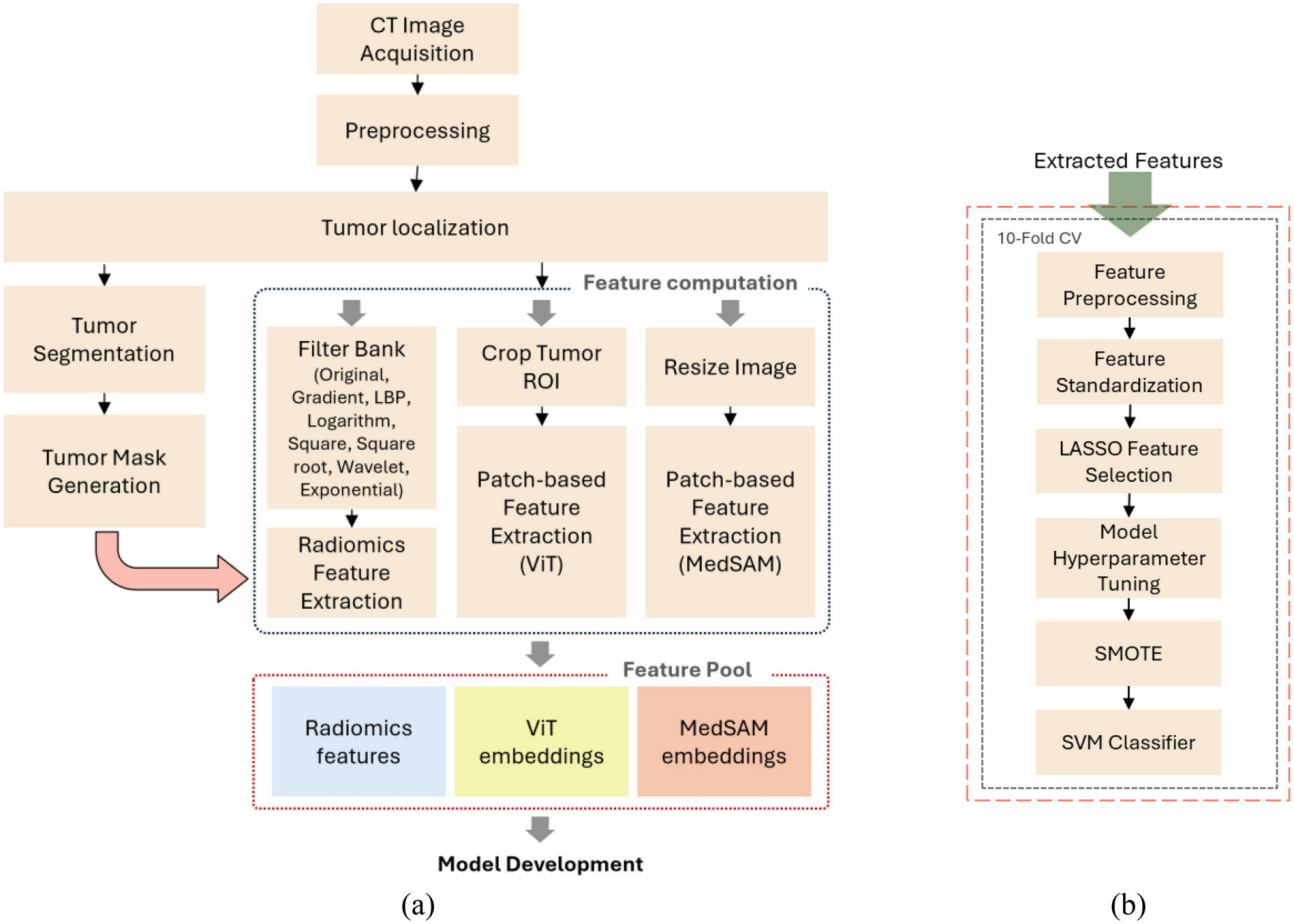
Flowchart of the developed models. **(a)** Feature extraction block diagram for both models. **(b)** Model development flowchart.

To analyze the effectiveness of features from radiomics alongside deep learning methods, we developed distinct machine learning models that utilized features from radiomics, ViT, MedSAM, and their integrations (Figure 3). For the integration models, we concatenated features from the different sources to form a unified feature vector. All models aimed to predict the 6-month PFS following chemotherapy treatment in OC patients.

Prior to model training, all preprocessing and modeling steps were performed within the inner loop of a nested, stratified cross-validation framework. All data-dependent procedures were fit exclusively on the training split and subsequently applied to the corresponding held-out data. Initially, features with zero variance were identified and removed (41). We then standardized features using z-score normalization (42). It transforms each feature *x* to have zero-mean and unit standard deviation according to z=(x−μ)/σ (where *z* is standardized feature value, *x* is the original feature value, μ and σ are mean and standard deviation computed on the training split). Next, we computed pairwise Pearson correlations and, for each highly correlated pair (above a predefined threshold of 0.95), removed one feature to reduce redundancy, improving generalization and reducing overfitting risk (43).

Considering the imbalance nature of the dataset, we applied the synthetic minority oversampling technique (SMOTE) on the training split inside the cross validation loop (44). SMOTE addresses class imbalance by generating synthetic samples for the minority class, thereby mitigating the risk of bias towards the majority class. This algorithm works by first selecting a non-dominant sample at random and then obtaining its k nearest neighbors (k = 5). Next, a specified number of new synthetic instances are created by interpolating between the minority sample and its neighbors in the feature space. By calculating the difference in distance between the feature vector and its neighbors, SMOTE synthesizes new instances that lie within the vicinity of the existing minority instances, effectively augmenting the minority class.

Next, dimensionality reduction was performed using the least absolute shrinkage and selection operator with cross-validation (LASSO-CV) method to form optimal feature ensemble. LASSO-CV is a modified version of the least squares regression technique incorporating L1 regularization to generate sparse coefficients for features (45). In this study, feature selection was carried out using Lasso CV from scikit-learn. Although the outcome variable was binary, labels were coded as 0/1 and LASSO was used solely as a sparsity-inducing feature selector, rather than as a probabilistic classifier. All features have been z-score standardized using training data prior to LASSO, and the regularization parameter λ was selected via the default 5-fold cross-validation within the training data by minimizing mean-squared error. In LASSO regression, the coefficients are estimated by minimizing $min_{\beta }\;\left(\frac{1}{2n}\sum_{i=1}^{n}{\left(y_{i}-\beta_{0}-\overset{m}{\underset{\;j=1}{\sum }}x_{ij}\beta_{j}\right)}^{2}\;\right)+\;\lambda \sum_{\;j=1}^{m}\left|\beta_{j}\right|$. Where β represents the feature coefficients, λ is the regularization parameter, *n* and *m* are number of samples and features, respectively. By setting some coefficients to zero, LASSO-CV effectively identifies and selects important features, thereby reducing the dimensionality of the dataset while preserving predictive accuracy (46). Features with non-zero coefficients were retained and passed to the downstream SVM classifier, enabling dimensionality reduction while improving interpretability and generalization (46).

Subsequently, we utilized a support vector machine (SVM) model (47) for predicting chemotherapy treatment responses, with hyperparameter tuning conducted via grid search cross validation (48). The main goal of the SVM classifier is to find an optimal hyperplane in high-dimensional space to separate different classes in the feature space (49). The hyperplane tries to maximize the margin between the closest samples of different classes. Hence, the optimization problem aims to minimize the $\frac{1}{2}w^{T}w+C\sum_{i=1}^{m}\zeta^{\left(i\right)}$ subject to $t^{\left(i\right)}(w^{T}x^{\left(i\right)}+b)\geq 1-\;\zeta^{\left(i\right)}$ and $\zeta^{\left(i\right)}\;\geq 0\;$ for i=1,2,…,m (47). *w* and *b* are normal vector and bias term, ζ denotes the slack variable, and *C* is a hyperparameter controlling the margin and adjusting coefficients for error optimization. Hyperparameters such as “C”, and “kernel” were optimized through an exhaustive search. Specifically, “C” values ranged from 0.1 to 10, and “kernel” options included “linear”, radial basis function' (RBF), and “polynomial”. For non-linear kernels we additionally tuned γ for RBF and the polynomial degree. The best configuration in each inner split was refit on the inner-training data and evaluated on the corresponding held-out fold.

Through model development and evaluation, we used a nested stratified cross-validation framework. The inner loop was repeated five times with stratified splits to reduce variability from random fold assignments and improve the stability of feature selection and hyperparameter tuning. Within each inner loop, feature preprocessing, SMOTE, feature selection, hyperparameter optimization, and SVM training were performed. The outer 10-fold loop was used exclusively for held-out testing to provide unbiased performance estimates. To enable probabilistic evaluation, SVM decision values were converted to class probabilities using Platt scaling (sigmoid calibration) by enabling *probability* *=* *True* in scikit-learn's SVC. Probability estimation was learned using training data only within the nested cross-validation procedure. Calibration curves and Brier scores were computed exclusively from predicted probabilities on the held-out outer test folds. No probability threshold optimization was performed on test folds; class labels were generated using the default SVM decision rule.

We evaluated the performance of the models using the receiver operating characteristic (ROC) curve (50), the area under the ROC curve (AUC) (51), and their respective confidence intervals (CIs). Additional metrics such as precision, recall, and F1-score were also assessed (52). Confidence intervals were computed via patient-level bootstrap (*N* = 200) on pooled out-of-fold predictions (53).

## 3 Results

**Figure 4:**
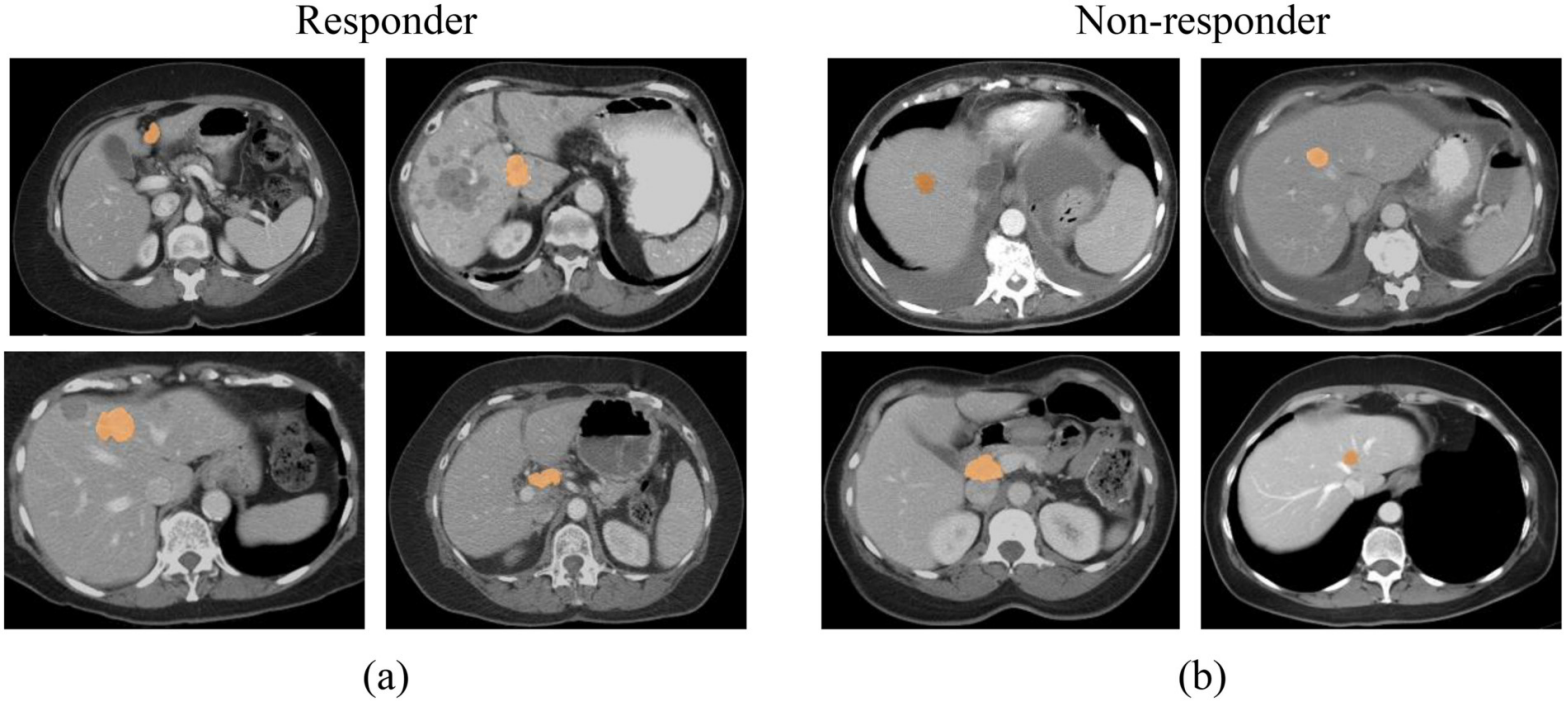
Sample CT images illustrate tumor segmentation. **(a)** Four images from the responder cohort, and **(b)** four images from the non-responder cohort.

Figures 4a,b present four sample CT images, representing both responder and non-responder cohorts. The segmented tumors in each image are highlighted in orange. Visual inspection of these images reveals no discernible features that differentiate between the responder and non-responder groups.

**Figure 5:**
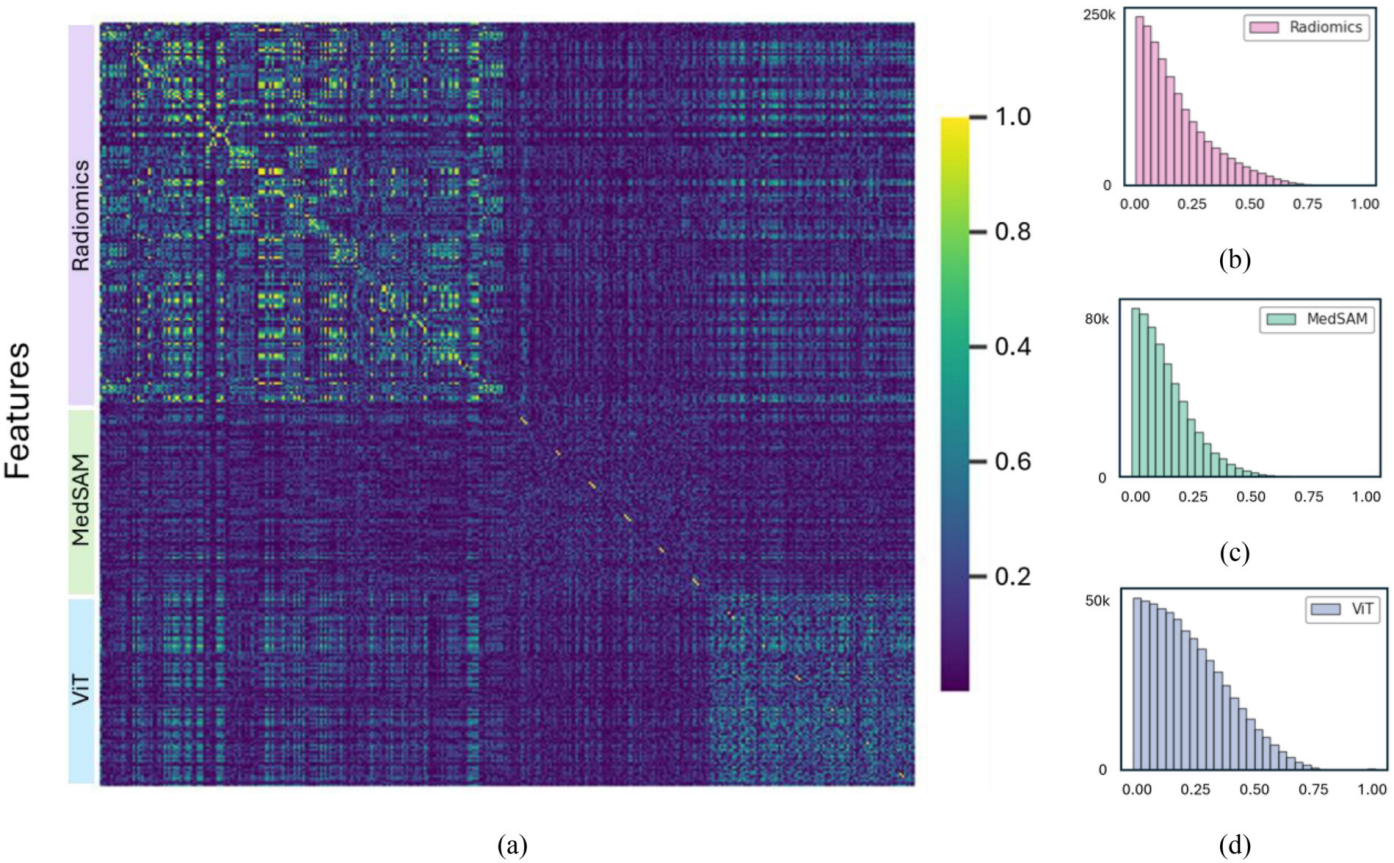
**(a)** Heatmap of Pearson correlation coefficients among all extracted features from radiomics, ViT-based, and MedSAM-based groups. **(b–d)** Individual heatmaps showing intra-group correlations.

Figure 5a displays heatmaps of the Pearson correlation coefficients among all extracted features from the radiomics, ViT-based, and MedSAM-based groups. The coefficients range from 0 to 1, with color intensity indicating the strength of correlation. As shown, while all feature groups demonstrate generally low internal dependency, the radiomics features exhibit higher intra-group correlations compared to those derived from ViT and MedSAM, as shown in Figures 5b–d. The inter-group correlations remain relatively low, suggesting that the different feature sets provide complementary information with minimal redundancy.

**Figure 6:**
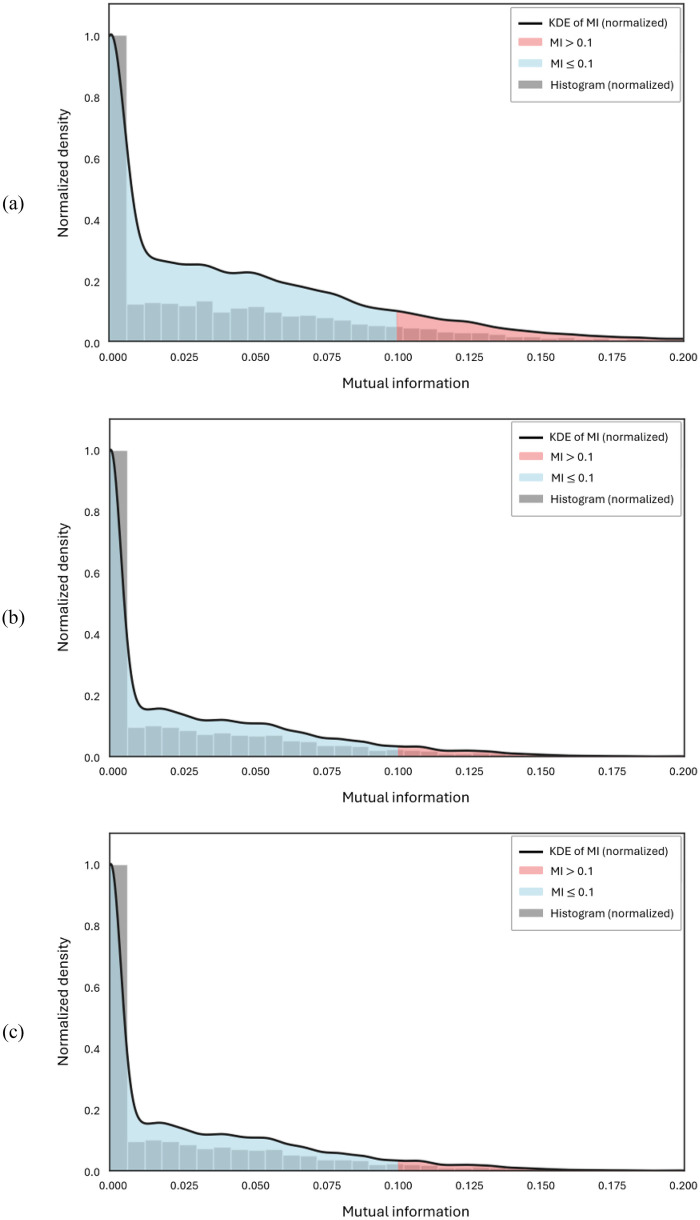
Normalized distributions of mutual information (MI) between feature pairs used in training multi-modal models. An MI threshold of 0.1 indicates minimal statistical dependency. **(a)** Radiomics and ViT features, **(b)** ViT and MedSAM features, and **(c)** Radiomics and MedSAM features.

To further examine the relationships among the selected feature groups in the context of integrated model development, Figure 6 presents the normalized distribution of mutual information (MI) between features employed in training the models. An MI threshold of 0.1 is used to indicate very low statistical dependency. As shown in Figure 6a, the distribution of MI values between the selected radiomic and ViT features reveals minimal redundancy, with over 90% of the values falling below the 0.1 threshold. Similar trends are observed in Figures 6b,c, corresponding to the ViT–MedSAM and radiomics–MedSAM combinations, respectively, suggesting consistently low inter-feature dependency across all integrated feature sets.

**Figure 7:**
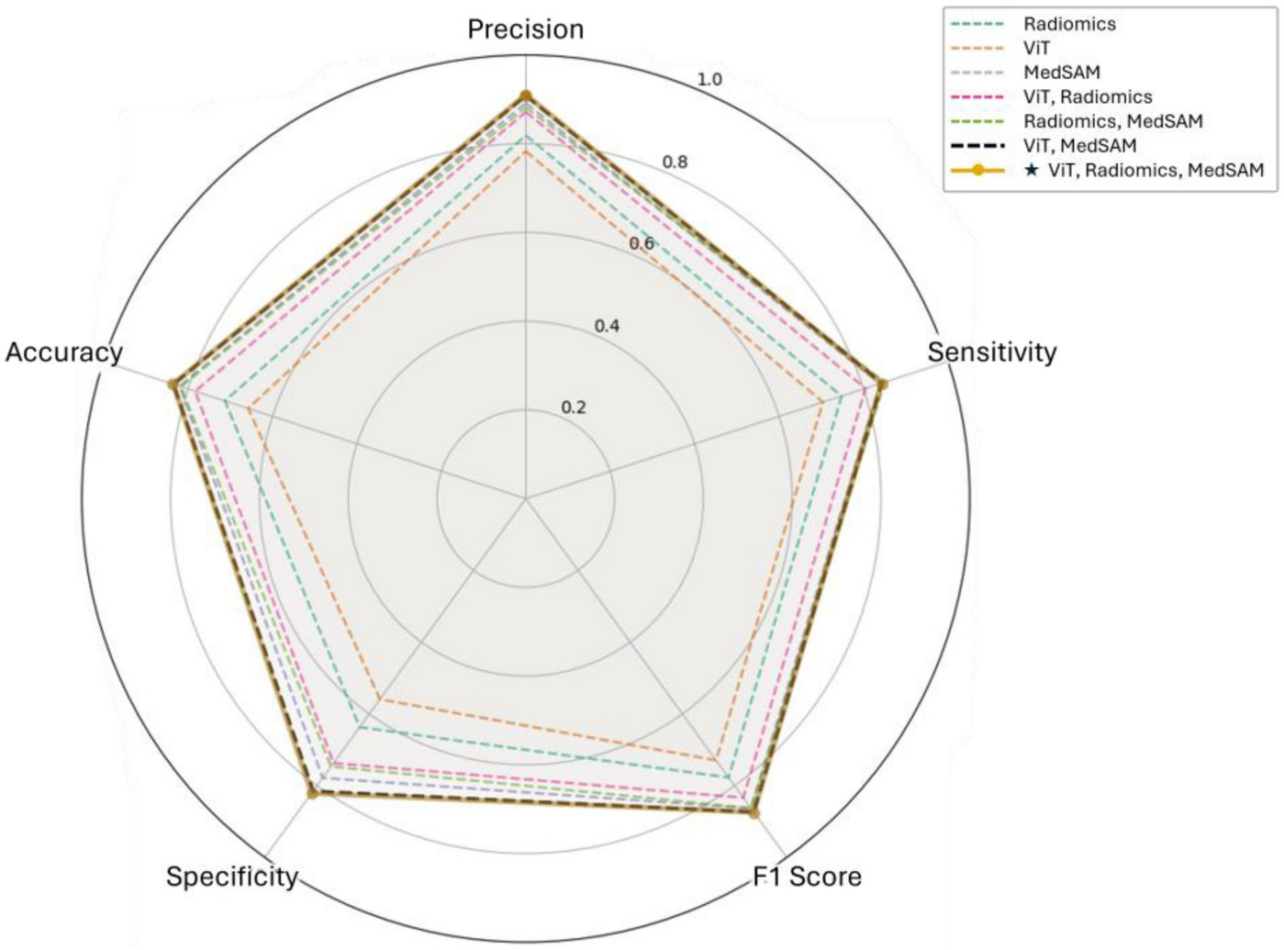
Radar chart comparing the performance of models trained on different feature sets across multiple evaluation metrics, including accuracy, precision, sensitivity, F1-score, and specificity.

Table 2 presents the performance of different feature sets and their combinations in predicting treatment response. Among individual modalities, MedSAM achieved the highest AUC (0.897 ± 0.034) and accuracy (0.808 ± 0.046), suggesting its effectiveness in capturing relevant imaging biomarkers. Radiomics and ViT, while lower performing individually, contributed to improved results when integrated. In the multi-representation setting, integrating all feature sets achieved the best performance (AUC 0.924 ± 0.037, accuracy 0.831 ± 0.042), indicating the complementary value of hand-crafted radiomics and deep embeddings. The ViT + MedSAM model reached a similar AUC (0.924 ± 0.032) and second-highest in accuracy (0.829 ± 0.039), reinforcing the advantage of multi-representation integration. According to the radar chart in Figure 7, which compares models across multiple evaluation metrics, similar trends are observed in precision, recall, F1-score, and specificity. Detailed values for performance metrics of each model are reported in Supplementary Table S1 (Supplementary Material). These results demonstrate the closely matched performance of the (ViT + MedSAM) and (ViT + Radiomics + MedSAM) models, further supporting the effectiveness of combining ViT and MedSAM features.

**Table 2:** Prediction performance of the models with confidence interval.

Features	AUC	Accuracy
Radiomics	0.780 ± 0.062	0.712 ± 0.044
ViT	0.679 ± 0.107	0.657 ± 0.056
MedSAM	0.897 ± 0.034	0.808 ± 0.046
Radiomics, ViT	0.865 ± 0.076	0.783 ± 0.045
Radiomics, MedSAM	0.890 ± 0.076	0.816 ± 0.050
ViT, MedSAM	**0.924** **± 0.032**	0.829 ± 0.039
Radiomics, ViT, MedSAM	**0.924** **± 0.037**	**0.831** **± 0.042**

Top-performing values for each metric are highlighted.

To assess the robustness of the reported performance to class imbalance handling, we additionally conducted a no-SMOTE ablation study for the best-performing integrated model (Radiomics + ViT + MedSAM). We used the same stratified nested cross-validation protocol with identical outer and inner folds, preprocessing, feature selection, and hyperparameter tuning. The full pipeline was repeated without applying SMOTE. As reported in Supplementary Table S2 in Supplementary Material, removal of SMOTE resulted in a modest reduction in performance (AUC 0.924 ± 0.037 with SMOTE vs. 0.908 ± 0.035 without SMOTE). Importantly, overall discriminative performance remained high, indicating that the observed performance is not driven by oversampling but reflects a robust predictive signal.

**Figure 8:**
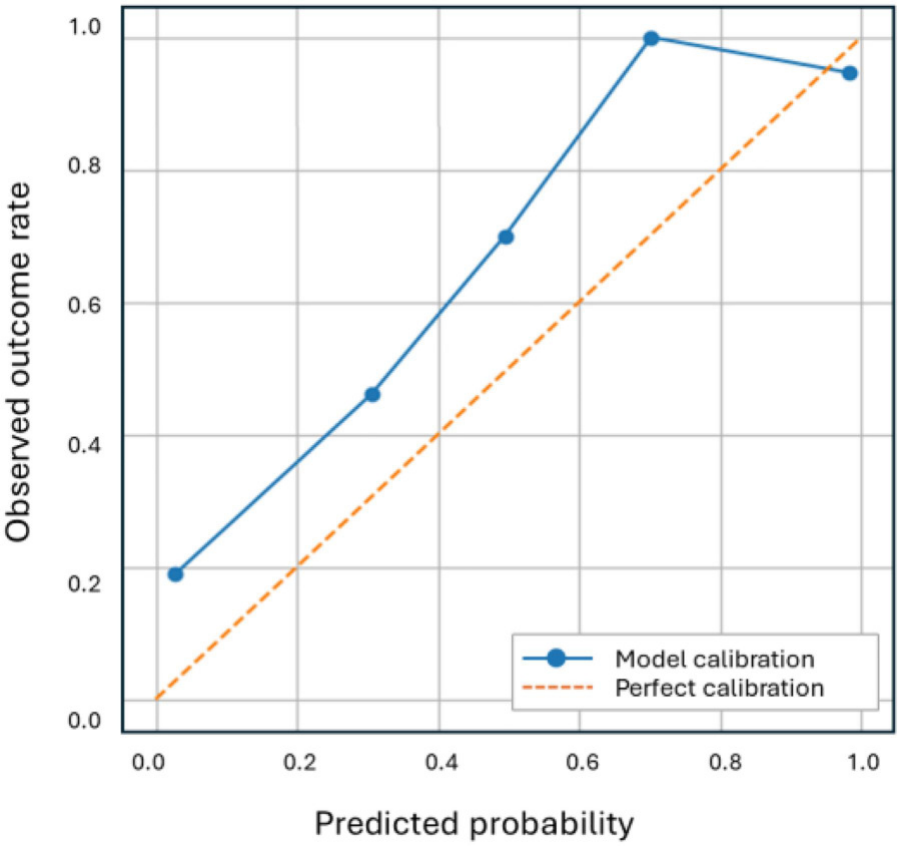
Calibration curve for the best performing model.

**Figure 9:**
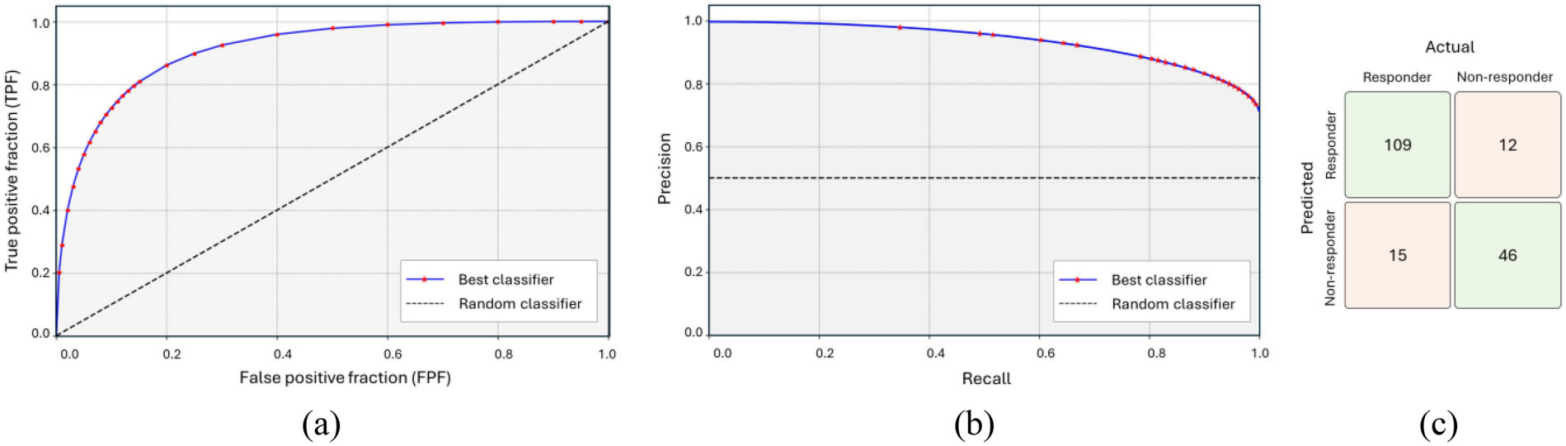
Performance of the six-month PFS prediction model achieved by the best performing classification model based on Radiomics, ViT and MedSAM feature integration. **(a)** ROC curve. **(b)** Precision-recall curve. **(c)** Confusion matrix.

Calibration analysis was conducted for the best-performing model. The calibration curve (Figure 8) demonstrates close alignment between predicted and observed event probabilities, indicating good probability reliability. The Brier score was 0.12, reflecting good overall calibration. Figure 9 presents the performance of our best-performing model. Figures 9a,b show the ROC and precision–recall (PR) curves with ROC-AUC of 0.92 and PR-AUC of 0.94. Figure 9c depicts the confusion matrices for that model, with a decision threshold set at 0.43 on the decision scores (selected by max-F1 on the training folds). The model correctly predicts 109 out of 124 responder cases and 46 out of 58 non-responder cases, resulting in a positive predictive value (PPV) of 90% and a negative predictive value (NPV) of 75%.

**Figure 10:**
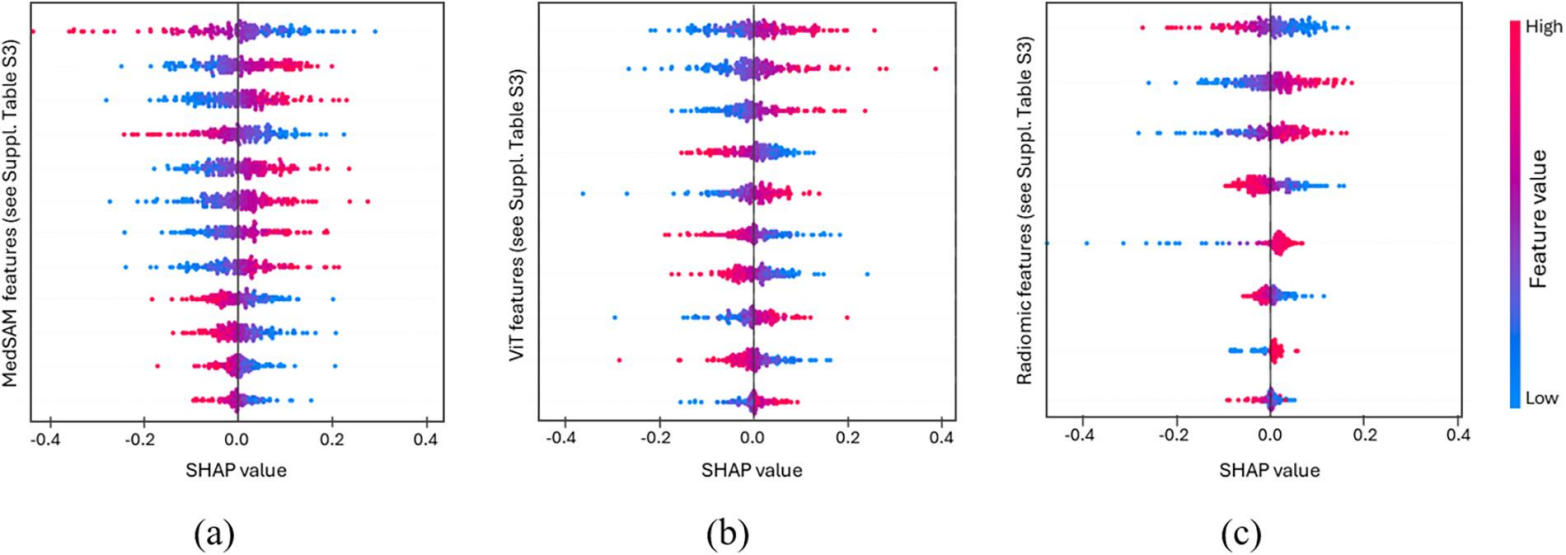
SHAP summary plots for the features selected in the best-performing model (Radiomics + ViT + MedSAM), shown separately by source: **(a)** MedSAM, **(b)** ViT, and **(c)** radiomics. To enhance visual clarity, feature names are not shown in the plots and are instead listed in Supplementary Table S3 in the Supplementary Material.

After LASSO feature selection, an average of approximately 30 features were retained per run. Figures 10a–c present SHAP (54) summary plots for the prognostic feature subsets (MedSAM, ViT, radiomics) used by the best-performing classifier. For visual clarity, the figures illustrate the relative importance of the selected features without explicit feature name annotations. The 30 features shown were taken from a representative run whose performance closely matched the cross-validated average. Among them, 12 originated from MedSAM, 10 from ViT, and 8 from radiomics. Feature identities and detailed descriptions are provided in Supplementary Table S3 in the Supplementary Material. Across cross-validation runs, feature attributions were highly stable, with the top 20 features consistently appearing in at least 80% of the outer folds. To summarize this consistency, the mean absolute SHAP values for these features across runs are provided in Supplementary Figure S1 in the Supplementary Material. This summary confirms that MedSAM-derived features exert the strongest influence on model predictions, while ViT-based and radiomic features also contribute meaningfully, though to a less extent.

## 4 Discussion

In this study, we investigated the value of multi-method imaging biomarkers for predicting chemotherapy response in ovarian cancer patients. Compared to previous research, our study has several unique characteristics.

First, radiomics features outperformed ViT features in this task, achieving an AUC of 0.780 ± 0.062 compared to 0.679 ± 0.107. This is probably why handcrafted radiomics features have the potential to identify and extract tumor-related information clearly, and they have been proven to have potentials in characterization of tumor phenotypes and prediction of outcomes in oncologic imaging (17). In contrast, although ViT models learn high-dimensional representations, they are pretrained primarily on non-medical datasets and therefore lack domain-specific medical knowledge. Nevertheless, their performance indicates the generalizability of transformer-based architectures in medical imaging applications. Prior CT radiomics studies in ovarian cancer have reported moderate-to-strong performance using handcrafted features and classical machine-learning pipelines (55–57); in comparison, our results suggest that integrating transformer-based embeddings provides additional prognostic signal beyond traditional radiomics alone.

Second, the MedSAM based model showed better results than the radiomics and ViT based models (Table 2), probably because it had been trained on extensive medical image datasets containing representations of clinical relevance. In particular, MedSAM by itself had a very good prediction performance, indicating that advanced foundation models already have a significant portion of the imaging signal that is typically captured by handcrafted radiomics encoded. Besides that, combining radiomics and ViT features led to an increased performance compared to each single-method that was used, which means that those features hold complementary information. Radiomics features provide interpretable, handcrafted descriptors of tumor intensity and texture, whereas ViT embeddings encode representations that capture more complex and subtle spatial patterns. The similar improvement in performance was shown through the combination of ViT and MedSAM features, which indicates that transformer models trained in different domains (natural vs. medical images) hold different, non-redundant information. Interestingly, adding radiomics features to the MedSAM-based model did not further improve performance, implying that MedSAM embeddings already encompass many structured tumor characteristics quantified by radiomics. In general, the results here suggest that multi-method fusion can capture a wider representation of tumor biology, which is reflected in the higher prediction performance of multi-method fusion models compared to single-method approaches. This conclusion is further supported by feature redundancy analyses, which revealed minimal correlation between ViT and MedSAM embeddings, consistent with their complementary nature.

Despite these promising results, several limitations should be acknowledged. First, the data used for this study was only from one institution, which may limit generalizability across scanners, acquisition protocols, and patient populations. To ensure the robustness and clinical transferability of the proposed approach, multi-center studies will be necessary in the future. Second, our feature fusion strategy relied on simple feature concatenation, which does not explicitly model interdependencies among representations; consequently, higher-dimensional or more discriminative feature sets may dominate the learning process, potentially diminishing the contribution of more subtle or indirect predictive information such as radiomics. Additionally, imaging features were extracted from a single axial slice with the largest tumor cross-section. While 3D may capture richer spatial information, this 2D approach reduced sensitivity to slice-thickness variability and segmentation uncertainty while maintaining strong predictive performance. Finally, this study focused exclusively on CT imaging and did not incorporate additional data sources such as histopathology, clinical variables, or genomics, which could further enhance predictive performance and model robustness. Despite these limitations, our findings provide meaningful insights into imaging-based prediction of chemotherapy response and advance the development of precision oncology strategies.

## Data Availability

The original contributions presented in the study are included in the article/Supplementary Material, further inquiries can be directed to the corresponding author/s.
