# Supplementary material for: A vision transformer-radiomics approach for enhanced chemotherapy outcome prediction in ovarian cancer
Source: Front Radiol. 2026 Mar 17;6:1702977. doi: 10.3389/fradi.2026.1702977 (PMC13036210; doi:10.3389/fradi.2026.1702977)
Supplement: Supplementary file 1 [file Datasheet1.pdf]

Table S1. Performance metrics of the models trained with different feature sets

Features	Accuracy	Precision	Sensitivity	F1 score	Specificity
Radiomics	0.71 ± 0.04	0.81 ± 0.03	0.74 ± 0.09	0.77 ± 0.04	0.63 ± 0.12
ViT	0.65 ± 0.05	0.78 ± 0.05	0.70 ± 0.14	0.72 ± 0.07	0.56 ± 0.17
MedSAM	0.80 ± 0.04	0.88 ± 0.03	0.83 ± 0.06	0.86 ± 0.03	0.77 ± 0.08
Radiomics, ViT	0.78 ± 0.04	0.87 ± 0.04	0.80 ± 0.07	0.83 ± 0.04	0.73 ± 0.13
Radiomics, MedSAM	0.81 ± 0.05	0.88 ± 0.04	0.84 ± 0.07	0.86 ± 0.04	0.74 ± 0.14
ViT, MedSAM	0.82 ± 0.03	0.90 ± 0.03	0.84 ± 0.06	0.87 ± 0.03	0.81 ± 0.06
Radiomics, ViT, MedSAM	0.83 ± 0.04	0.91 ± 0.03	0.84 ± 0.05	0.87 ± 0.03	0.82 ± 0.06

Table S2. No-SMOTE ablation results for the best-performing integrated model (Radiomics + ViT + MedSAM) using the same stratified nested cross-validation protocol

Features	AUC	Accuracy	Precision	Sensitivity	F1 score	Specificity
Radiomics, ViT, MedSAM (Best-performing)	0.90 ± 0.03	0.81 ± 0.04	0.89 ± 0.03	0.84 ± 0.06	0.86 ± 0.03	0.79 ± 0.06

Table S3. List and description of features selected by the best-performing integrated model (Radiomics + ViT + MedSAM). Features are grouped by source (MedSAM, ViT, and radiomics). Within each group, features are ordered from top to bottom according to decreasing mean absolute SHAP value, consistent with the feature importance ranking shown in Figure 10

MedSAM	ViT	Radiomics
449	236	Wavelet-HLH_GLSZM_SizeZoneNonUniformityNormalized
705	154	Wavelet-LHL_GLDM_DependenceEntropy
247	22	Wavelet-HHH_GLDM_LargeDependenceEmphasis
499	503	Wavelet-LHH_GLSZM_GrayLevelVariance
649	466	Wavelet-LHL_firstorder_Skewness
260	329	Logarithm_GLSZM_SmallAreaEmphasis
463	704	LBP_firstorder_10Percentile
71	591	Squareroot_GLDM_DependenceVariance
455	385	-
491	674	-
145	-	-
549	-	-

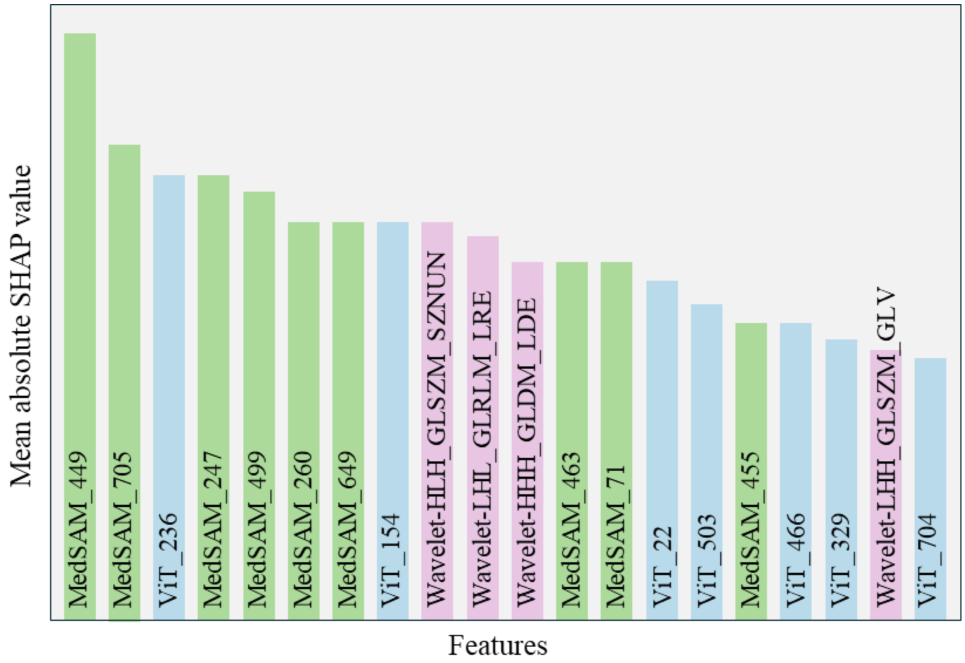

Figure 1. Contribution of the top 20 optimal feature set to the best-performing classifier as quantified by SHAP
